# Multiple co-occurring genetic variants in an elderly patient with Liddle’s-like syndrome: a case report and literature review

**DOI:** 10.1186/s13256-026-06473-2

**Published:** 2026-08-25

**Authors:** Yunlong Liang, YangZhi Zheng, Hua Yuan, Li Guo, Xinping Wang, Jing Zhang, Juan Wang, Yanhai Zhang, Linan Duan

**Affiliations:** 1 Department of Geriatrics, Xijing Hospital 986 Hospital, The Fourth Military Medical University, Xi’an, 710002 Shaanxi China; 2 Department of Cardiovascular Medicine, Xijing Hospital, The Fourth Military Medical University, Xi’an, 710004 Shaanxi China; 3 Department of Neurosurgery, Xijing Hospital 986 Hospital, The Fourth Military Medical University, Xi’an, 710002 Shaanxi China

**Keywords:** Liddle's-like syndrome, Refractory hypokalemia, Hypertension, Multiple genetic variants, Case report

## Abstract

**Background:**

Liddle syndrome (LS) is a rare autosomal dominant condition classically defined by early-onset hypertension, hypokalemia, metabolic alkalosis, and a suppressed renin-aldosterone profile, primarily driven by gain-of-function mutations in the *SCNN1A*, *SCNN1B*, or *SCNN1G* genes encoding the epithelial sodium channel (ENaC). Nevertheless, genetically investigated cases exhibiting Liddle’s-like phenotypes in the elderly population remain exceptionally rare.

**Methods:**

We report a 61-year-old male admitted with recurrent limb weakness and resistant hypertension. A comprehensive clinical workup, including biochemical profiles, RAAS evaluation, and 24-h ABPM, was conducted to exclude common secondary causes. Following informed consent, whole-exome sequencing (WES) was performed, with candidate variants validated by Sanger sequencing and assessed per ACMG/AMP guidelines. To guide therapeutic management, a narrative literature review of similar cases was also performed searching PubMed, Web of Science, and CNKI using keywords like "Liddle syndrome," "elderly," "hypertension," and "hypokalemia.".

**Results:**

Admission tests showed hypokalemia (2.94 mmol/L), compensated metabolic alkalosis, and suppressed renin-aldosterone levels. 24-h ABPM indicated severe blood pressure fluctuations up to 185/120 mmHg. Genetic testing revealed three heterozygous variants of uncertain significance (VUS)—*CYP3A5* (c.1253 + 83C > T), *SCNN1G* (c.1727A > G, p.Lys576Arg), and *WNK1* (c.5636A > T, p.Asp1879Val)—without any known pathogenic variants. A diagnostic trial with triamterene (50 mg twice daily) restored normal potassium levels (> 3.5 mmol/L). After a 30-day combination therapy of controlled-release nifedipine and irbesartan, blood pressure stabilized between 110 and 133 and 69-85 mmHg.

**Conclusion:**

We report an elderly patient with chronic hypokalemia and resistant hypertension. After ruling out common secondary causes (for example, primary aldosteronism, Cushing's syndrome, pheochromocytoma), Liddle's-like syndrome was clinically diagnosed based on the low-renin/low-aldosterone biochemical phenotype, compensated metabolic alkalosis, literature review, and a selective response to triamterene. This case suggests that Liddle's-like syndrome must be considered in the differential diagnosis of hypokalemic hypertension in older adults to ensure early and targeted therapy.

**Supplementary Information:**

The online version contains supplementary material available at https://doi.org/10.1186/s13256-026-06473-2.

## Introduction

LS was initially described in 1963 by Liddle *et al.* in a Caucasian pedigree featuring a 16-year-old proband [1]. Classically, the disease manifests in adolescence or early adulthood, characterized by early-onset hypertension, hypokalemia, and the suppression of both plasma renin activity (PRA) and aldosterone secretion. As its clinical presentation mimics primary aldosteronism yet remains refractory to mineralocorticoid receptor antagonists, LS is frequently termed "pseudoaldosteronism" [2]. The current cornerstone of pharmacological management consists of ENaC inhibitors, notably amiloride or triamterene.

Current research confirms that LS is a rare, autosomal dominant monogenic form of hypertension predominantly driven by gain-of-function mutations in the *SCNN1A*, *SCNN1B*, or *SCNN1G* genes—encoding the α, β, and γ subunits of the ENaC [3]. These genetic aberrations provoke constitutive ENaC overactivation, thereby inducing persistent sodium reabsorption across the renal distal tubules and collecting ducts [4–6]. The advent of advanced genetic sequencing has facilitated the early recognition and diagnosis of a growing cohort of adolescent and young adult LS patients [7, 8]. Although this progress has heightened clinical suspicion in younger populations, it has arguably entrenched the paradigm of LS as a uniquely early-onset pathology, resulting in its premature categorization as an age-specific condition.

Conversely, literature documenting Liddle's-like syndrome—characterized by hypokalemic hypertension coupled with a suppressed renin-aldosterone profile—in the geriatric population remains exceedingly scarce [9–11]. Delayed diagnosis and suboptimal management in this demographic markedly increase the risk of devastating cardiovascular and cerebrovascular events, notably stroke, ultimately undermining their quality of life and long-term prognosis [12]. Herein, we report the case of an elderly patient presenting with long-standing hypokalemia and resistant hypertension, in whom a clinical diagnosis of possible Liddle's-like syndrome was established following a rigorous exclusion of alternative secondary causes. This report serves to underscore the necessity of incorporating Liddle's-like syndrome into the differential diagnosis of hypokalemic hypertension in older adults, ensuring prompt therapeutic intervention and the averting of life-threatening sequelae.

## Case presentation

A 61-year-old male was admitted with long-standing hypokalemia and resistant hypertension. His clinical history dates back 20 years when he first presented to Shaanxi Provincial People's Hospital with sudden lower limb weakness, where an initial diagnosis of hypokalemia (exact values unrecorded) was made, and symptoms resolved following intravenous potassium replacement. Strikingly, his hypokalemia-induced fatigue was persistent rather than paroxysmal. Over a 20-year span, his serum potassium consistently remained below 3.5 mmol/L. During this time, he irregularly self-medicated with oral potassium chloride solution (10 mL/dose) to manage recurrent fatigue without monitoring his potassium levels. The weakness manifested only during severe hypokalemic episodes and was partially, but never fully, alleviated by oral supplementation. Crucially, his serum potassium had virtually never reached a normal range over the past two decades. Furthermore, he was diagnosed with Grade 3 hypertension five years ago (peak blood pressure: 180/80 mmHg), which remained refractory to 30 mg/day of nifedipine controlled-release tablets. One week before admission, due to progressively worsening fatigue, outpatient laboratory tests at a community hospital revealed severe hypokalemia (K^+^: 2.94 mmol/L), with unremarkable sodium (140.1 mmol/L), chloride (100.2 mmol/L), and calcium (1.17 mmol/L) levels.

Upon admission, physical examination demonstrated elevated blood pressure at 150/70 mmHg, with notable diastolic lability ranging from 61 to 120 mmHg, and a body mass index (BMI) of 22.4 kg/m^2^. Cardiopulmonary, abdominal, and neurological examinations were unremarkable. Diagnostic workup revealed a complete blood count (WBC: 4.98 × 10^9^/L, ANC: 3.24 × 10^9^/L, RBC: 4.29 × 10^12^/L, Hb: 144 g/L, MCV: 96.5 fL, PLT: 139 × 10^9^/L) and comprehensive serum chemistry (urea: 5.3 mmol/L, creatinine: 85 μmol/L, uric acid: 523 μmol/L, triglycerides: 5.08 mmol/L, cystatin C: 1.61 mg/L, eGFR (CKD-EPI): 84.81 mL/min/1.73 m^2^, chloride: 101.0 mmol/L, magnesium: 0.95 mmol/L, calcium: 2.27 mmol/L, phosphorus: 1.12 mmol/L; liver panel: TBIL: 21.6 μmol/L, DBIL: 5.8 μmol/L, IBIL: 15.8 μmol/L, AST: 11.4 U/L, ALT: 4 U/L, ALP: 72 U/L, GGT: 62 U/L, total protein: 64.7 g/L, albumin: 41.2 g/L, globulin: 23.5 g/L). Urinalysis showed microalbumin of 0.15 g/L and specific gravity of 1.015; 24-h urine protein excretion was 0.11 g/24 h (UPCR < 0.2 g/gCr). Stool routine, coagulation profiles, and thyroid, and pituitary function tests were unremarkable. Of note, during the initial phase of hospitalization before the renin-aldosterone profile was obtained, the patient was empirically administered two doses of spironolactone. This was paradoxically followed by worsening muscular weakness, a precipitous fall in serum potassium to a critical threshold of 2.3 mmol/L, and a transient blood pressure spike up to 182/120 mmHg. His clinical condition stabilized after spironolactone was promptly withdrawn and therapy with oral potassium chloride solution and nifedipine CR tablets was instituted. Key laboratory parameters pertinent to the diagnosis of LS are summarized in Table 1.

**Table 1:** Pertinent laboratory parameters

Analyte	Value	Normal reference range ^a^
pH	7.44	7.35 ~ 7.45
BE (mmol/L)	2.9	− 3 ~ + 3
HCO_3_⁻ (mmol/L)	27.4 ↑	22 ~ 26
K⁺ (mmol/L)	2.94 ↓	3.5 ~ 5.5
Na⁺ (mmol/L)	143.0	136 ~ 146
Urinary pH	6.5	4.5 ~ 8.0
Hcy (μmol/L)	26.6 ↑	< 15
Uric Acid (μmol/L)	523 ↑	208 ~ 428
Glucose (mmol/L)	8.88 ↑	3.9 ~ 6.1
HbA1c (%)	5.9	4.0—6.0
Total Cholesterol (mmol/L)	5.24 ↑	< 5.18
Triglycerides (mmol/L)	8.35 ↑	< 1.7
24-h Urinary Potassium Excretion (mmol/24 h)	8.2 ↓	25 ~ 100
24-h Urinary Sodium Excretion (mmol/24 h)	104.4 ↓	130 ~ 260
Urinary 17-Ketosteroids (mg/24 h)	3.6 ↓	5 ~ 30
Urinary 17-Hydroxycorticosteroids (mg/24 h)	1.9 ↓	2 ~ 10
Aldosterone (pg/mL)	24.7 ↓	69 ~ 296
DRC (μIU/mL)	0.88 ↓	42 ~ 45.6

*BE* Base Excess, *Hcy* Homocysteine, *DRC* Direct Renin Concentration

^a^Normal reference value in Xijing Hospital 986 Hospital

A 24-h ambulatory blood pressure monitoring (ABPM) revealed labile hypertension, with blood pressure fluctuating between 136/61 and 185/120 mmHg. Echocardiography demonstrated: interventricular septal thickness of 10 mm; left ventricular posterior wall thickness of 10 mm; left ventricular internal dimension of 50 mm; left atrial transverse systolic diameter of 34 mm; right atrial transverse systolic diameter of 29 mm; right ventricular transverse diastolic diameter of 21 mm; and left ventricular dimensions (anteroposterior, transverse, longitudinal in systole/diastole) of 41/54 mm, 36/50 mm, and 76/87 mm, respectively. The left ventricular ejection fraction (LVEF) was 52.75%, with a fractional shortening (FS) of 26.46%. Left ventricular wall motion analysis indicated hypokinesis below the chordae tendineae level in the inferior and posterior walls (excursion reduced by ~ 3–4 mm) with increased myocardial echogenicity, as well as hypokinesis of the anterior wall below the lower papillary muscle level (excursion reduced by ~ 4 mm); thickness and wall motion of the remaining free walls were normal. The right coronary cusp of the aortic valve showed mild thickening and increased echogenicity, with diastolic regurgitation (regurgitant gap ~ 2 mm). Color Doppler Flow Imaging (CDFI) revealed a vena contracta width of ~ 3 mm, jet length of 7.6 cm, jet area of 9.3 cm^2^, and regurgitant volume of 10 mL. The position, thickness, elasticity, echogenicity, and excursion of other valves were unremarkable. CDFI showed mild mitral regurgitation (jet length: 2.2 cm, area: 2.1 cm^2^, volume: 2 mL) and tricuspid regurgitation (jet length: 1.3 cm, Vmax: 223 cm/s, PGmax: 20 mmHg; estimated pulmonary artery systolic pressure: 25 mmHg). Pulsed-wave Doppler at the mitral inflow recorded a diastolic forward flow pattern with an E peak velocity of 58 cm/s and an A peak velocity of 87 cm/s (E/A ratio < 1).

Vascular evaluation via carotid Doppler ultrasonography revealed atherosclerosis in the right subclavian artery and bilateral common/internal carotid arteries, accompanied by an elevated resistive index (RI) in the vertebral arteries. Renal artery Doppler showed an increased RI in bilateral renal arteries, despite normal vascular diameters and flow velocities, consistent with hypertensive nephrosclerosis or microvascular remodeling secondary to chronic hypertension. Subsequent abdominal ultrasonography and CT confirmed intact bilateral adrenal glands, but identified a right renal cyst and a left renal microcalculus (Fig. S1). Other abdominal ultrasound findings included mild splenomegaly, with normal structures in the liver, gallbladder, and pancreas. Ophthalmic examination indicated mild bilateral bulbar conjunctival hyperemia and localized temporal subconjunctival hemorrhage in the left eye. Other ancillary evaluations included sinus rhythm with non-specific T-wave changes on electrocardiogram (ECG) and normal thyroid parenchyma on thyroid ultrasonography.

Whole-exome sequencing revealed three VUS with partial phenotypic concordance: a *CYP3A5* variant associated with susceptibility to salt-sensitive hypertension, a *SCNN1G* variant linked to LS type 2 (autosomal dominant pseudohypoaldosteronism type IB3) and bronchiectasis spectrum disorders, and a *WNK1* variant implicated in hereditary sensory and autonomic neuropathy type II and pseudohypoaldosteronism type IIC (see Supplementary Methods). The variants were identified through clinical whole-exome sequencing (BGI Genomics, Shenzhen; Report No. DX1616), as summarized in Table 2. All variants were classified in accordance with ACMG/AMP guidelines [13–15]. Detailed ACMG/AMP scoring criteria are provided in Tables 1, 2 and 3 of the supplementary materials.

**Table 2:** Whole-exome sequencing results

Gene	Associated Disorder(s)/Inheritance	Genomic Position	Transcript	Nucleotide Change (Protein Change)	Exon/ Intron	Genotype	ACMG/AMP Classification
*CYP3A5*	Susceptibility to primary salt-sensitive hypertension	chr7:992,500 93	NM_000777.5	c 1253 + 83 C > T	Intron 11	Heterozygous	PP3
*SCNN1* *G*	Liddle syndrome type 2 Autosomal dominant pseudohypoaldosteronism type 1B3 Bronchiectasis spectrum disorders	chr16:23,226 567	NM_001039.4	c 1727 A > G(p.Lys 576Arg)	Exon 13	Heterozygous	BP4 (Moderate) PM2 (Supporting)
*WNK1*	Hereditary sensory-autonomic neuropathy type II Pseudohypoaldosteronism type IIC	chr12:10,052 89	NM_018979.4	c 5636 A > T(p.Asp 1879Val)	Exon 24	Heterozygous	PM2 (Supporting) BP4

*ACMG* American College of Medical Genetics and Genomics. *AMP* Association for Molecular Pathology

No pathogenic or likely pathogenic variants related to the current phenotype were detected; all findings were limited to VUS

*WES was conducted by BGI Genomics (Shenzhen, China; Report No.: DX1616)

**Table 3:** Clinical and genetic features of reported cases of liddle's-like syndrome

Author	Subjects	Key Clinical Features		Family history	ENaC gene	Comorbidities	Therapeutic drug	Efficacy and prognosis
		Hypertension&Hypokalemia	Low renin & aldosterone, metabolic alkalosis					
Kashif Nadeem M[9]	88‐year‐old	Pos	Pos	No	Neg	Alzheimer’s dementia, Hypercholesterolemia	Amiloride	Improved
Pepersack T[10]	83‐year‐old	Pos	Pos	No	Neg	Depression, Type 2 diabetes mellitus, Congestive heart failure	Triamterene	Improved
Yamaguchi E[11]	65-year-old	Pos	Pos	No	Neg	Membranous nephropathy, Hyperlipidemia, Gastric cancer	Triamterene	After total gastrectomy, Triamterene D/C
Suman S [16]	60-year-old	Pos	Pos	Yes	Pos	Not reported	Amiloride	Improved
Present case	61-year-old	Pos	Pos	No	Pos	Type 2 diabetes	Triamterene	Improved

*Pos.* Positive, *Neg.* Negative, *D/C* Discontinue

**Fig. 1:**
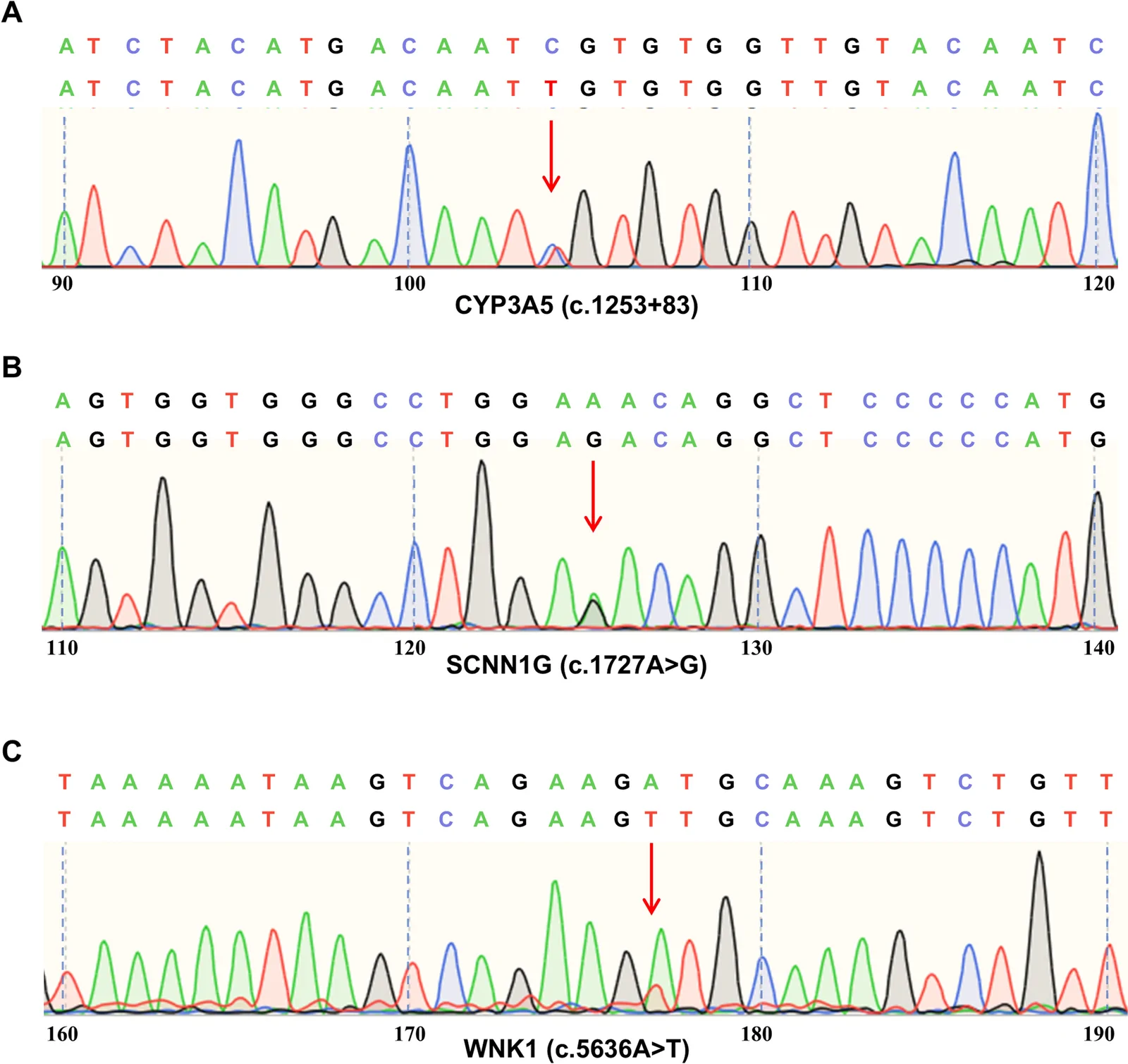
Results of Sanger sequencing. Sanger sequencing identified mutations in **A** *CYP3A5* (intronic, c.1253 + 83), **B** *SCNN1G* (exon 13, codon 1727 missense), and **C** *WNK1* (exon 24, codon 5636 missense). Sequencing was performed by Tsingke Biotechnology Co., Ltd

In this patient, the following variants were identified: a *CYP3A5* c.1253 +  83 C > T intronic variant, a *SCNN1G* c.1727A > G (p.Lys576Arg) missense variant in exon 13, and a *WNK1* c.5636A > T (p.Asp1879Val) missense variant in exon 24 (Fig. 1).

Three candidate variants were identified through clinical whole-exome sequencing (performed by BGI Shenzhen; Report No. DX1616). In silico functional characterization was conducted using *SIFT* (predicting missense pathogenicity), SpliceAI (predicting splice-altering effects), and PhyloP Vertebrates (assessing evolutionary conservation). The *CYP3A5* variant was predicted by SpliceAI to be damaging, suggesting a potential impact on pre-mRNA splicing, despite residing in a non-conserved region. In contrast, the *SCNN1G* variant was consistently classified as benign across multiple predictors (*SIFT*, MutationTaster, SpliceAI) and also localizes to a non-conserved region. For the *WNK1* variant, conflicting predictions were obtained: *SIFT* and MutationTaster indicated pathogenicity, whereas SpliceAI and dbscSNV suggested neutrality. All variants are located in non-conserved genomic regions, which may attenuate their putative functional impacts. These computational predictions offer preliminary insights into the potential pathogenicity of these variants; however, further functional validation is required. A comprehensive summary of these predictions is provided in Supplementary Table 4.

**Fig. 2:**
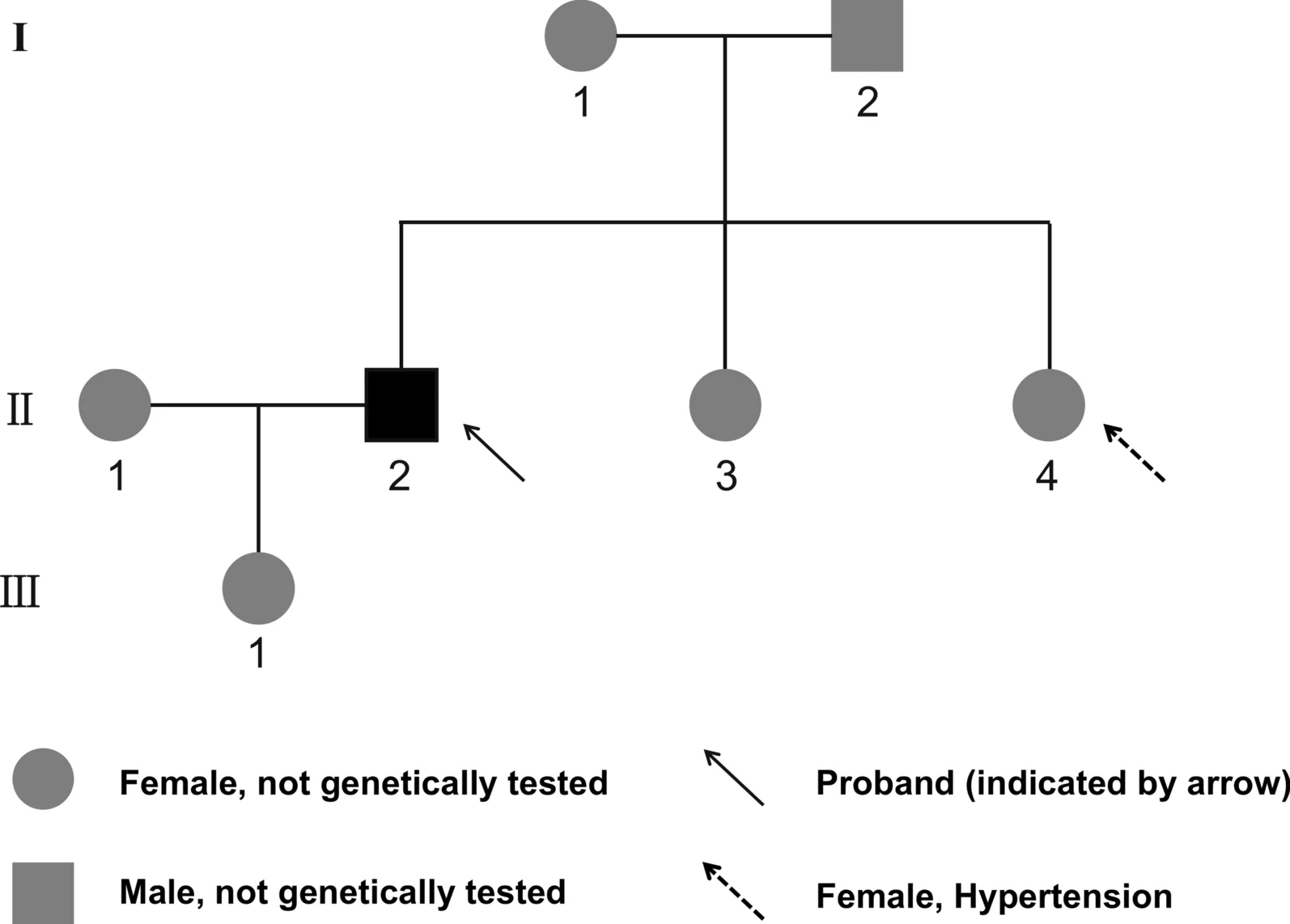
Pedigree of the patient

In terms of family history, one of the patient's two elder sisters had a history of hypertension in the absence of hypokalemic symptoms; parental medical history was unavailable (Pedigree, Fig. 2). The patient explicitly denied chronic consumption of licorice, diuretics, laxatives, glucocorticoids, herbal supplements, or other potentially toxic agents. Subsequent follow-up assessments confirmed the absence of hypertension or hypokalemia in the proband's other siblings and offspring. Due to the unavailability of parental samples, familial co-segregation analysis could not be performed. While the *SCNN1G* variant merits further investigation in the context of hypokalemia, and the *CYP3A5* variant has documented literature associations with salt-sensitive hypertension, the absence of functional assays and co-segregation data precludes definitive confirmation of the direct pathogenicity of these VUS in our patient.

Although the patient's clinical phenotype strongly indicated Liddle's-like syndrome, genetic sequencing failed to identify definitive pathogenic ENaC variants. Regarding the differential diagnosis, primary aldosteronism was ruled out due to the marked suppression of both plasma renin and aldosterone. Apparent mineralocorticoid excess (AME) was excluded based on normal urinary 17-hydroxycorticosteroids and 17-ketosteroids, absence of licorice exposure, and blood gas evidence of compensated metabolic alkalosis. Congenital adrenal hyperplasia (CAH) was discounted given normal urinary steroid metabolites and no history of virilization or precocious puberty. Cushing's syndrome was clinically excluded by the absence of typical cushingoid signs. Gordon syndrome (pseudohypoaldosteronism type II) was inconsistent with the presence of metabolic alkalosis rather than acidosis, alongside suppressed renin. Activating mutations of the mineralocorticoid receptor (Geller syndrome) were ruled out by suppressed aldosterone levels and spironolactone inefficacy. Finally, pheochromocytoma was excluded by normal bilateral adrenal morphology on abdominal CT.

To navigate this diagnostic challenge and inform our therapeutic strategy, we performed a non-systematic literature review across PubMed, Web of Science, and CNKI (up to July 31, 2025). Employing search terms including "Liddle syndrome," "elderly," "hypertension," and "hypokalemia," we targeted genetically profiled patients aged ≥ 60 years exhibiting Liddle or Liddle's-like features (hypertension, hypokalemia, and a suppressed renin-aldosterone axis). This search identified a mere four relevant case reports (Table 3). Strikingly, three of these cases displayed classic Liddle's-like biochemical profiles and responded excellently to triamterene or amiloride, despite lacking mutations in the ENaC β or γ subunit genes [9–11, 16]. The sole genetically corroborated case was a 60-year-old male presenting with resistant hypertension, hypokalemia, metabolic alkalosis, and a positive family history, driven by a novel *SCNN1G* mutation [16]. Collectively, these findings underscore that patients with low-renin/low-aldosterone hypokalemia and metabolic alkalosis may still derive significant therapeutic benefit from ENaC inhibitors, even without established ENaC mutations. Guided by this evidence, we instituted a diagnostic-therapeutic trial of triamterene, establishing a practical basis for our treatment decision.

Based on the literature evidence, a comprehensive pharmacological regimen was initiated. The initial treatment for refractory hypokalemia included triamterene (50 mg BID) and oral potassium chloride supplementation. Resistant hypertension was concurrently managed with nifedipine controlled-release (30 mg/day) and irbesartan (150 mg/day). Given the newly diagnosed type 2 diabetes mellitus, dapagliflozin (10 mg/day) was prescribed. Furthermore, the patient received targeted treatment for concurrent metabolic disorders, including febuxostat (40 mg daily) for hyperuricemia, fenofibrate (100 mg tid) for hypertriglyceridemia, and folic acid (0.8 mg daily) for hyperhomocysteinemia.

**Fig. 3:**
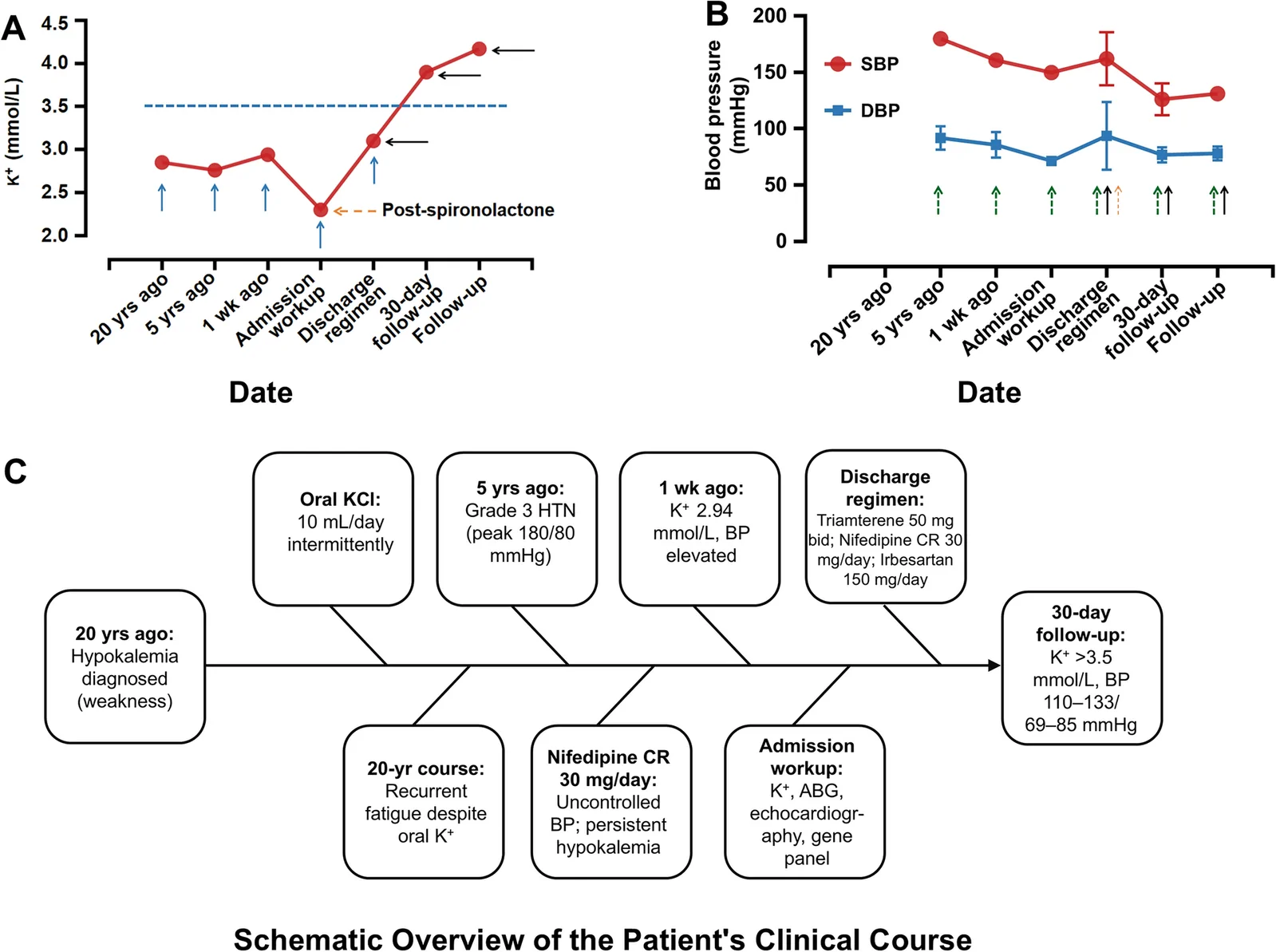
Schematic overview of the patient's clinical course. **A** Serum potassium curve (blue arrows: oral KCl solution; black arrows: oral triamterene; brown dashed arrow: Post-spironolactone). **B** Blood pressure curve before and after triamterene and nifedipine CR treatment (green arrows: oral nifedipine CR; black arrows: triamterene; brown arrows: irbesartan). **C** Simplified clinical treatment timeline

At the 30-day follow-up, normokalemia (> 3.5 mmol/L) was successfully established. Consequently, the patient was weaned off oral potassium chloride and transitioned to long-term maintenance therapy with triamterene (50 mg BID) (Fig. 3A). Hemodynamic stability was sustained, with blood pressure ranging from 110–133/69–85 mmHg, effectively managed by 30 mg/day of nifedipine CR (Fig. 3B). Glycemic control remained optimal (fasting glucose: 4–7 mmol/L; postprandial glucose: 5–10 mmol/L), and the chronic fatigue was completely abated. Reflecting on the patient’s 20-year medical history, his intractable hypokalemia and hypertension demonstrated remarkable improvement following the diagnostic-therapeutic trial with the triamterene and nifedipine CR combination (Fig. 3C).

## Discussion

The present case exhibits a clinical profile distinctively divergent from classic Liddle syndrome: (1) late-onset manifestation, with hypertension diagnosed only at age 56; (2) a profound temporal dissociation characterized by roughly 15 years of isolated hypokalemia preceding the hypertension; (3) an inappropriately low 24-h urinary potassium excretion (8.2 mmol/24 h), contradicting the anticipated renal potassium wasting paradigm; and (4) a divergent therapeutic response to triamterene, yielding rapid normokalemia but only partial blood pressure abatement. Furthermore, genetic sequencing unveiled three VUS in the *SCNN1G*, *CYP3A5*, and *WNK1* genes. We emphasize that, lacking functional assays for these VUS, our objective is to postulate the underlying pathophysiological mechanisms of this atypical phenotype rather than asserting direct pathogenicity. Consequently, this case is most cautiously categorized as a Liddle's-like syndrome.

### Temporal dissociation of hypokalemia and hypertension

Classically, the constitutive activation of ENaC in Liddle syndrome orchestrates enhanced sodium reabsorption in the distal nephron [17]. This generates an amplified luminal electronegativity that drives potassium efflux, accompanied by volume-expanded hypertension. Thus, hypokalemia and hypertension classically co-occur early in life. The paradox in our patient—where isolated hypokalemia predated the onset of hypertension by nearly two decades—challenges the paradigm of sheer ENaC overactivation. Informed by the literature, we hypothesize two underlying mechanisms. First, assuming the *SCNN1G* variant confers only a mild functional alteration, the resulting subtle shift in the distal tubular electrochemical gradient could provoke detectable potassium wasting without precipitating a massive sodium retention-volume expansion cascade. Hypokalemia would thereby serve as an earlier and more sensitive sentinel phenotype. Second, chronic normotension may have been sustained through robust physiological compensations, including enhanced renal medullary blood flow, an upregulated pressure-natriuresis response, and profound RAAS suppression. Eventually, age-related arterial stiffening and the concomitant exhaustion of these compensatory reserves unmasked the chronic volume load, translating it into clinical hypertension. Third, during the initial phase of hospitalization, empirical administration of two doses of spironolactone provoked a worsening of fatigue, a precipitous drop in serum potassium to a critical threshold of 2.3 mmol/L, and a transient blood pressure spike up to 182/120 mmHg. Spironolactone failed to alleviate the hypokalemia and paradoxically aggravated it. Upon establishing the low-renin, low-aldosterone profile, we deduced that the core pathophysiological driver was intrinsic ENaC overactivation rather than mineralocorticoid receptor hyperactivation. As a mineralocorticoid receptor antagonist, spironolactone was inherently ineffective and likely exacerbated potassium depletion through volume-mediated shifts. These findings further substantiate the hypothesis that ENaC pathway dysregulation existed early in the disease trajectory as a sustained driver of hypokalemia, while the delayed emergence of hypertension signifies the gradual breakdown of volume-regulatory compensatory mechanisms.

### Inappropriately low 24-h urinary potassium excretion

In classical paradigms of ENaC-driven renal potassium wasting, urinary potassium excretion is characteristically augmented [18]. In stark contrast, our patient exhibited an inappropriately low 24-h urinary potassium of 8.2 mmol/day, a discrepancy that ostensibly undermines the diagnosis of renal potassium wasting. A plausible physiological explanation posits that during urine collection, the patient was in a state of profound systemic potassium depletion (serum K^+^: 2.94 mmol/L) (Fig. 3A). In such critical hypokalemic states, robust compensatory mechanisms may maximally upregulate potassium reabsorption in the proximal tubule and the loop of Henle as a life-sustaining salvage pathway, yielding a pseudonormalized or paradoxically attenuated urinary potassium output [19]. This physiological adaptation is well-documented in severe hypokalemia. The lack of follow-up 24-h urinary potassium data post-normokalemia constitutes a salient limitation, directly obscuring the definitive validation of ENaC-mediated renal potassium wasting. Additionally, episodic potassium loss, urine collection errors, or occult gastrointestinal losses cannot be definitively excluded. Although clinical evaluations effectively ruled out overt gastrointestinal causes like vomiting and diarrhea, this specific laboratory finding constitutes a notable phenotypic limitation, challenging a purely ENaC-centric mechanistic interpretation.

### Integrated interpretation of the three VUS

*SCNN1G* encodes the ENaC γ subunit; thus, this VUS serves as the most direct genetic tether to the ENaC pathway [20–23]. The prompt normalization of potassium following triamterene administration provides circumstantial pharmacological evidence of ENaC overactivation, ostensibly supporting the functional relevance of this variant. Nevertheless, this fails to satisfy the rigorous functional criteria of the ACMG guidelines required to upgrade a VUS to a likely pathogenic status. Notably, while the *SCNN1G* p.Lys576Arg variant in our patient lies outside the canonical PY motif (amino acids 623–627), its adjacent proximity to this critical C-terminal regulatory domain is noteworthy [24–26]. Furthermore, *CYP3A5* mediates renal cortisol metabolism, potentially modulating local ligand availability for the mineralocorticoid receptor. Conversely, *WNK1* is a pivotal kinase orchestrating the downstream SPAK-NCC axis; its perturbation could dysregulate the sodium-chloride cotransporter in the distal convoluted tubule. In the present case, the *WNK1* variant may drive an atypical phenotype through a pleiotropic ligand/kinase regulatory network, or simply act as a genetic background modifier. To date, the phenotypic linkage of these three VUS remains purely conjectural. We postulate that this multigenic signature establishes a susceptible genetic background, predisposing the individual to environmental triggers or age-related physiological decline, thereby collectively forging this late-onset, mild, and phenotypically overlapping atypical Liddle's-like state. Importantly, absent validation from channel electrophysiology in heterologous expression systems and protein interaction assays, this hypothesis represents an integrative conceptual framework rather than an established pathogenic mechanism.

### Cardiac structural and functional remodeling

The echocardiographic findings—specifically the hypokinesis of the left ventricular inferior and posterior walls, alongside the quantitative thickening of the interventricular septum and posterior wall—can be partially attributed to the dual pressure and volume overload imposed by chronic hypertension and aortic regurgitation. Concurrently, given the patient's age and hypertensive history as established risk factors, underlying ischemic heart disease cannot be excluded. Moreover, profound chronic hypokalemia intrinsically disrupts cardiomyocyte electrophysiology and contractility, which may culminate in reversible focal wall motion abnormalities. The non-specific T-wave abnormalities on the ECG further corroborate the coexistence of electrolyte derangements and hypertensive heart disease. Therefore, we recommend repeat cardiac imaging following the establishment of sustained normokalemia to assess the reversibility of the myocardial dysfunction.

An elevated serum homocysteine level (26.6 μmol/L) was noted in the patient. This moderate hyperhomocysteinemia was likely driven by advanced age, male sex, and long-standing hypertension with renal microvascular impairment, as implied by an increased renal resistive index. While not a classical hallmark of Liddle or Liddle's-like syndrome, hyperhomocysteinemia constitutes an independent cardiovascular risk factor that potentially aggravated the observed hypertensive vascular remodeling and echocardiographic changes. Accordingly, folic acid supplementation was prescribed for cardiovascular risk reduction. Considering the ongoing debate surrounding the clinical efficacy of homocysteine-lowering interventions, this concurrent finding is best interpreted as a prognostic modifier rather than an integral component of the primary pathophysiology.

### Dissociated clinical response to triamterene

Administration of the epithelial sodium channel inhibitor triamterene prompted rapid normokalemia, definitively pointing to ENaC-dependent potassium hypersecretion in the distal nephron. Conversely, blood pressure achieved only modest reduction, a dichotomy potentially driven by two factors: First, years of poorly controlled hypertension might have induced irreversible arterial remodeling and fixed systemic vascular resistance, rendering the sole relief of ENaC-mediated volume expansion inadequate for complete blood pressure normalization. Second, parallel ENaC-independent pro-hypertensive pathways may be at play. For example, if the *WNK1* variant subtly upregulates NCC activity and enhances sodium reabsorption in the distal convoluted tubule, thiazide diuretics might represent a more mechanistically targeted antihypertensive option. This clinical inference, derived from the divergent therapeutic responses, establishes a verifiable hypothesis for tailored pharmacological management and prospective functional validation.

### Limitations

Several limitations of the present study must be acknowledged. First, all identified variants are VUS. The absence of in vitro functional assays and familial co-segregation data precludes definitive assertions of pathogenicity. While the pedigree illustrates the clinical phenotypes of the proband and select relatives, missing genotypic data obscures the variants' true co-segregation patterns. Second, the single 24-h urinary potassium measurement introduces uncertainty; the lack of a repeat assessment following normokalemia restoration undermines the robustness of the renal potassium wasting diagnosis. Third, owing to the retrospective design, certain clinical evaluations—such as follow-up echocardiography post-potassium correction and coronary angiography—are incomplete. Fourth, the incorporated literature review is a non-systematic narrative summary unaligned with PRISMA guidelines, inherently subjecting it to potential selection bias and limiting its utility to background context. Finally, standardized evaluations of triggering factors, including precise dietary sodium intake and licorice exposure, were not conducted. Consequently, future functional investigations and comprehensive family genetic evaluations are indispensable next steps.

## Conclusion

In conclusion, we report an elderly patient with chronic hypokalemia and resistant hypertension. Based on laboratory findings of low renin, low aldosterone, and compensated metabolic alkalosis, Liddle's-like syndrome was highly suspected. Genetic testing revealed three VUS in *SCNN1G*, *CYP3A5*, and *WNK1*, but none have proven pathogenicity. The patient showed a selective response to triamterene: blood potassium quickly returned to normal, but blood pressure only partially improved. The unusual features of this case show that when genetic testing does not provide a clear diagnosis, a diagnostic-therapeutic trial of triamterene is valuable for both treatment and reverse diagnosis. This case provides a useful reference for diagnosing and treating elderly patients with resistant hypertension and hypokalemia.

## Supplementary Information

Supplementary Material 1.

## Data Availability

No datasets were generated or analysed during the current study.

## Abbreviations

LS: Liddle syndrome
ACMG: American College of Medical Genetics and Genomics
AMP: Association for Molecular Pathology
ENaC: Epithelial sodium channel
PAC: Plasma aldosterone concentration
CT: Computed tomography
VUS: Variants of uncertain significance
WES: Whole-exome sequencing
PRA: Plasma renin activity
DRC: Direct Renin Concentration
BMI: Body mass index
ABPM: Ambulatory blood pressure monitoring
LVEF: Left ventricular ejection fraction
FS: Fractional shortening
CDFI: Color Doppler Flow Imaging
RI: Resistive index
ECG: Electrocardiogram

## Declarations

### Patient perspective

Upon diagnosis, the patient expressed great relief after two decades of unexplained symptoms. He is committed to adhering to the new treatment regimen and hopes his case can help others.
