# Supplementary material for: Multiple co-occurring genetic variants in an elderly patient with Liddle’s-like syndrome: a case report and literature review
Source: J Med Case Rep. 2026 Aug 25;20:733. doi: 10.1186/s13256-026-06473-2 (PMC13760772; doi:10.1186/s13256-026-06473-2)
Supplement: Supplementary file 1 [file 13256_2026_6473_MOESM1_ESM.docx]

**Fig. S1. Non-contrast CT images of the adrenal glands.**

(A) Right adrenal gland; (B) Left adrenal gland. The adrenal glands are indicated by red boxes.

**Supplementary Methods: Whole-Exome Sequencing and Bioinformatics Analysis**

1. DNA Extraction and Whole Exome Sequencing

Genomic DNA was extracted from 5 mL of the proband's peripheral blood using a commercial DNA extraction kit (MagPure Buffy Coat DNA Midi KF Kit, Magen, Guangzhou, China). DNA quality and concentration were assessed using a spectrophotometer (NanoDrop 2000, Thermo Fisher, USA). Genomic DNA was fragmented using Fragmentase (BGI), and the resulting fragments were size-selected using magnetic beads. The selected fragments underwent end repair, 'A'-tailing, amplification, and purification to construct the sequencing library. Exonic regions and adjacent splice sites were captured and enriched using the KAPA HyperExome probe set (Roche, USA). The constructed libraries were assessed for fragment size and concentration using an Agilent 2100 Bioanalyzer and BMG. The pooled libraries were then circularized into single-stranded DNA circles and sequenced on the high-throughput DNBSEQ-T7 sequencing platform, generating 100 bp paired-end reads. The sequencing quality control metrics were as follows: average sequencing depth of the target region ≥ 200X, with >98.5% of target bases covered at >20X.

2. Bioinformatic Analysis of Sequencing Data

The raw sequencing data (FASTQ files) were processed through the following bioinformatic pipeline:

a. Quality Control: FastQC software was used to perform quality control on the raw reads to assess sequencing quality.
b. Alignment: BWA (Burrows-Wheeler Aligner) software was used to align the filtered reads to the human reference genome (GRCh37/hg19).
c. BAM File Processing: The generated Sequence Alignment/Map (SAM) files were converted to Binary Alignment/Map (BAM) format, sorted, and indexed using SAMtools. PCR duplicates were marked using Picard Tools.
d. Variant Calling: Single Nucleotide Variants (SNVs) and small Insertions/Deletions (Indels) were identified using the Genome Analysis Toolkit (GATK) HaplotypeCaller, following GATK best practices. Exome-level Copy Number Variants (CNVs) were detected using ExomeDepth.

3. Variant Filtering and Interpretation Pipeline

Given the "proband-only" study design, a stringent, stepwise filtering strategy was employed to prioritize potential candidate variants from the initial large set of called variants (typically tens of thousands).

Step 1: Basic Quality and Impact Filtering

Variants were annotated using bcfanno for information including genomic coordinates, gene, Human Genome Variation Society (HGVS) nomenclature, and functional consequence. Synonymous variants and intronic variants (except those affecting splice sites) were filtered out. Public databases, including dbSNP, the NHLBI GO Exome Sequencing Project (ESP6500), the Exome Aggregation Consortium (ExAC), and the 1000 Genomes Project (1000 GP), were used to filter out variants with a minor allele frequency (MAF) > 0.005 (0.5%).

Step 2: *In silico* Prediction

The filtered missense variants were assessed for potential deleteriousness using prediction software SIFT, MutationTaster, and Condel. Splice site disruption was predicted using SpliceAI and dbscSNV. Nucleotide conservation was assessed using PhyloP Vertebrates, PhyloP Placental Mammals, and GERP++.

Step 3: Candidate Gene Selection and Interpretation

Disease association was annotated using databases including Online Mendelian Inheritance in Man (OMIM), the Human Gene Mutation Database (HGMD), and ClinVar. Based on the above filtering and correlation with the patient's clinical phenotype, candidate pathogenic gene mutations were selected. Given the patient's presentation with refractory hypokalemia and hypertension, priority was given to variants in genes known to be associated with relevant pathways, such as blood pressure regulation and renal salt handling (e.g., genes encoding ENaC subunits SCNN1A, SCNN1B, SCNN1G; WNK kinase genes WNK1, WNK4; genes involved in aldosterone synthesis and action).

Step 4: Pathogenicity Assessment

The final candidate variants were evaluated according to the standards and guidelines from the American College of Medical Genetics and Genomics (ACMG) and the Association for Molecular Pathology (AMP). At this stage, only three variants of uncertain significance (VUS) in the CYP3A5, SCNN1G, and WNK1 genes, which showed partial phenotypic consistency, were retained for further reporting and discussion. All other variants were filtered out due to high frequency in control populations, benign in silico predictions, or lack of a plausible association with the patient's phenotype.

**ACMG/AMP Classification**

**Supplementary Table 1.**

Criteria for Classifying Pathogenic Variants

| **Very strong evidence of pathogenicity.** | |
| --- | --- |
| PVS1 | Null variant (nonsense, frameshift, canonical +/−1 or 2 splice sites, initiation codon, single or multi-exon deletion) in a gene where loss of function (LOF) is a known mechanism of disease |
| **Strong evidence of pathogenicity.** | |
| PS1 | Same amino acid change as a previously established pathogenic variant  regardless of nucleotide change  Example: Val->Leu caused by either G>C or G>T in the same codon  Caveat: Beware of changes that impact splicing rather than at the amino acid/protein level |
| PS2 | De novo (both maternity and paternity confirmed) in a patient with the  disease and no family history  Note: Confirmation of paternity only is insufficient. Egg donation, surrogate  motherhood, errors in embryo transfer, etc. can contribute to non-  Maternity |
| PS3 | Well-established in vitro or in vivo functional studies supportive of a  damaging effect on the gene or gene product  Note: Functional studies that have been validated and shown to be  reproducible and robust in a clinical diagnostic laboratory setting are  considered the most well-established |
| PS4 | The prevalence of the variant in affected individuals is significantly  increased compared to the prevalence in controls  Note 1: Relative risk (RR) or odds ratio (OR), as obtained from case-control  studies, is >5.0 and the confidence interval around the estimate of RR or OR  does not include 1.0. See manuscript for detailed guidance.  Note 2: In instances of very rare variants where case-control studies may  not reach statistical significance, the prior observation of the variant in  multiple unrelated patients with the same phenotype, and its absence in  controls, may be used as moderate level of evidence. |
| **Moderate evidence of pathogenicity** | |
| PM1 | Located in a mutational hot spot and/or critical and well-established  functional domain (e.g. active site of an enzyme) without benign variation |
| PM2 | Absent from controls (or at extremely low frequency if recessive) (see Table 6)  in Exome Sequencing Project, 1000 Genomes or ExAC  Caveat: Population data for indels may be poorly called by next generation  sequencing |
| PM3 | For recessive disorders, detected in trans with a pathogenic variant  Note: This requires testing of parents (or offspring) to determine phase |
| PM4 | Protein length changes due to in-frame deletions/insertions in a non-repeat  region or stop-loss variants |
| PM5 | Novel missense change at an amino acid residue where a different  missense change determined to be pathogenic has been seen before  Example: Arg156His is pathogenic; now you observe Arg156Cys  Caveat: Beware of changes that impact splicing rather than at the amino  acid/protein level |
| PM6 | Assumed de novo, but without confirmation of paternity and maternity |
| **Supporting evidence of pathogenicity.** | |
| PP1 | Co-segregation with disease in multiple affected family members in a gene  definitively known to cause the disease |
| PP2 | Missense variant in a gene that has a low rate of benign missense variation  and where missense variants are a common mechanism of disease |
| PP3 | Multiple lines of computational evidence support a deleterious effect on  the gene or gene product (conservation, evolutionary, splicing impact, etc)  Caveat: As many in silico algorithms use the same or very similar input for  their predictions, each algorithm should not be counted as an independent  criterion. PP3 can be used only once in any evaluation of a variant. |
| PP4 | Patient’s phenotype or family history is highly specific for a disease with a  single genetic etiology |
| PP5 | Reputable source recently reports variant as pathogenic but the evidence is  not available to the laboratory to perform an independent evaluation |

*Source: Reference *Richards S et al* [1].

**Supplementary Table 2.**

Criteria for Classifying Benign Variants

| **Stand-Alone evidence of benign impact.** | |
| --- | --- |
| BA1 | Allele frequency is above 5% in Exome Sequencing Project, 1000 Genomes,  or ExAC |
| **Strong evidence of benign impact.** | |
| BS2 | Observed in a healthy adult individual for a recessive (homozygous),  dominant (heterozygous), or X-linked (hemizygous) disorder with full  penetrance expected at an early age |
| BS3 | Well-established in vitro or in vivo functional studies shows no damaging  effect on protein function or splicing |
| BS4 | Lack of segregation in affected members of a family  Caveat: The presence of phenocopies for common phenotypes (i.e. cancer,  epilepsy) can mimic lack of segregation among affected individuals. Also,  families may have more than one pathogenic variant contributing to an  autosomal dominant disorder, further confounding an apparent lack of  segregation. |
| **Supporting evidence of benign impact.** | |
| BP1 | Missense variant in a gene for which primarily truncating variants are  known to cause disease |
| BP2 | Observed in trans with a pathogenic variant for a fully penetrant dominant  gene/disorder; or observed in cis with a pathogenic variant in any  inheritance pattern |
| BP3 | In-frame deletions/insertions in a repetitive region without a known  Function |
| BP4 | Multiple lines of computational evidence suggest no impact on gene or  gene product (conservation, evolutionary, splicing impact, etc)  Caveat: As many in silico algorithms use the same or very similar input for  their predictions, each algorithm cannot be counted as an independent  criterion. BP4 can be used only once in any evaluation of a variant. |
| BP5 | Variant found in a case with an alternate molecular basis for disease |
| BP6 | Reputable source recently reports variant as benign but the evidence is not  available to the laboratory to perform an independent evaluation |
| BP7 | A synonymous (silent) variant for which splicing prediction algorithms  predict no impact to the splice consensus sequence nor the creation of a  new splice site AND the nucleotide is not highly conserved |

*Source: Reference *Richards S et al.* [1].

**Supplementary Table 3**

Rules for Combining Criteria to Classify Sequence Variants

| **Pathogenic** | |
| --- | --- |
| 1. | 1 Very Strong (PVS1) AND |
|  | a. ≥1 Strong (PS1–PS4) OR |
|  | b. ≥2 Moderate (PM1–PM6) OR |
|  | c. 1 Moderate (PM1–PM6) and 1 Supporting (PP1–PP5) OR |
|  | d. ≥2 Supporting (PP1–PP5) |
| 2. | ≥2 Strong (PS1–PS4) OR |
| 3. | 1 Strong (PS1–PS4) AND |
|  | a. ≥3 Moderate (PM1–PM6) OR |
|  | b. 2 Moderate (PM1–PM6) AND ≥2 Supporting (PP1–PP5) OR |
|  | c. 1 Moderate (PM1–PM6) AND ≥4 Supporting (PP1–PP5) |
| **Likely Pathogenic** | |
| 1. | 1 Very Strong (PVS1) AND 1 Moderate (PM1–PM6) OR |
| 2. | 1 Strong (PS1–PS4) AND 1–2 Moderate (PM1–PM6) OR |
| 3. | 1 Strong (PS1–PS4) AND ≥2 Supporting (PP1–PP5) OR |
| 4. | ≥3 Moderate (PM1–PM6) OR |
| 5. | 2 Moderate (PM1–PM6) AND ≥2 Supporting (PP1–PP5) OR |
| 6. | 1 Moderate (PM1–PM6) AND ≥4 Supporting (PP1–PP5) |
| **Benign** | |
| 1. | 1 Stand-Alone (BA1) OR |
| 2. | ≥2 Strong (BS1–BS4) |
| **Likely Benign** | |
| 1. | 1 Strong (BS1–BS4) and 1 Supporting (BP1–BP7) OR |
| 2. | ≥2 Supporting (BP1–BP7) |

*Variants should be classified as Uncertain Significance if other criteria are unmet or the criteria for benign and pathogenic are contradictory.

*Source: Reference *Richards S et al.* [1].

**Supplementary Table 4**

In silico pathogenicity prediction of identified variants

| Gene | Missense Prediction | | | Splice Site Prediction | | | Conservation Scores | | |
| --- | --- | --- | --- | --- | --- | --- | --- | --- | --- |
|  | *SIFT* | *Mutation*  *Taster* | *Condel* | *SpliceAI* | *dbscSNV_*  *RF* | *bscSNV_*  *ADA* | *PhyloP*  *Vertebrates* | *PhyloP*  *Placetal*  *Mammals* | *GERP++* |
| *CYP3A5* | N/A | N/A | N/A | Damaging | N/A | N/A | Non-conserved | Non-conserved | N/A |
| *SCNN1*  *G* | Tolerated | Polymorphism | Neutral | Tolerated | N/A | N/A | Non-conserved | Non-conserved | Non-conserved |
| *WNK1* | Damaging | Disease causing | Neutral | Tolerated | N/A | N/A | Non-conserved | Non-conserved | Non-conserved |

Supplementary Table 4. In silico pathogenicity prediction of identified variants. Predictions were performed using multiple computational tools to assess the potential functional impact of missense and splice site variants identified through whole-exome sequencing (WES). WES was conducted by BGI Genomics (Shenzhen, China; Report No. : DX1616). SIFT: Predicts whether an amino acid substitution affects protein function. “Tolerated” indicates a neutral effect; “Damaging” suggests a deleterious effect. MutationTaster: Assesses the disease-causing potential of DNA alterations. “Polymorphism” implies a benign variant; “Disease causing” indicates pathogenicity. SpliceAI: Predicts the impact of variants on pre-mRNA splicing. “Neutral” suggests no splice effect; “Damaging” indicates a potential splice alteration. dbscSNV_RF & dbscSNV_ADA: Two ensemble-based methods for predicting splice site variants. Scores are not available (N/A) for non-splice-related variants. PhyloP (Vertebrates/Placental Mammals): Measures evolutionary conservation. “Non-conserved” indicates low sequence constraint across species. GERP++: Estimates nucleotide-level evolutionary constraint. “Non-conserved” suggests a lack of functional conservation. Note: “N/A” indicates that no prediction was available or applicable for the given variant type. Variants in non-conserved regions may have reduced likelihood of functional impact.

**References**

[1] Richards S, Aziz N, Bale S, Bick D, Das S, Gastier-Foster J, Grody WW, Hegde M, Lyon E, Spector E, Voelkerding K, Rehm HL; ACMG Laboratory Quality Assurance Committee. Standards and guidelines for the interpretation of sequence variants: a joint consensus recommendation of the American College of Medical Genetics and Genomics and the Association for Molecular Pathology. Genet Med. 2015 May;17(5):405-24. https://doi.org/10.1038/gim.2015.30. Epub 2015 Mar 5.
